# Monitoring of nighttime EEG slow‐wave activity during dexmedetomidine infusion in patients with hyperactive ICU delirium: An observational pilot study

**DOI:** 10.1111/aas.14131

**Published:** 2022-08-23

**Authors:** Tero Ala‐Kokko, Kristo Erikson, Juha Koskenkari, Jouko Laurila, Jukka Kortelainen

**Affiliations:** ^1^ Division of Intensive Care Medicine, Research Group of Surgery, Anesthesiology, and Intensive Care Medicine Oulu University Hospital and Medical Research Center Oulu Finland; ^2^ Physiological Signal Analysis Team, Center for Machine Vision and Signal Analysis University of Oulu and Medical Research Center Oulu Finland; ^3^ Cerenion Oy Oulu Finland

**Keywords:** C‐trend, RCSQ, sleep

## Abstract

**Background:**

The disturbance of sleep has been associated with intensive care unit (ICU) delirium. Monitoring of EEG slow‐wave activity (SWA) has potential in measuring sleep quality and quantity. We investigated the quantitative monitoring of nighttime SWA and its association with the clinical evaluation of sleep in patients with hyperactive ICU delirium treated with dexmedetomidine.

**Methods:**

We performed overnight EEG recordings in 15 patients diagnosed with hyperactive delirium during moderate dexmedetomidine sedation. SWA was evaluated by offline calculation of the C‐Trend Index, describing SWA in one parameter ranging 0 to 100 in values. Average and percentage of SWA values <50 were categorized as poor. The sleep quality and depth was clinically evaluated by the bedside nurse using the Richards‐Campbell Sleep Questionnaire (RCSQ) with scores <70 categorized as poor.

**Results:**

Nighttime SWA revealed individual sleep structures and fundamental variation between patients. SWA was poor in 67%, sleep quality (RCSQ) in 67%, and sleep depth (RCSQ) in 60% of the patients. The category of SWA aligned with that of RCSQ‐based sleep quality in 87% and RCSQ‐based sleep depth in 67% of the patients.

**Conclusion:**

Both, SWA and clinical evaluation suggested that the quality and depth of nighttime sleep were poor in most patients with hyperactive delirium despite dexmedetomidine infusion. Furthermore, the SWA and clinical evaluation classifications were not uniformly in agreement. An objective mode such as practical EEG‐based solution for sleep evaluation and individual drug dosing in the ICU setting could offer potential in improving sleep for patients with delirium.

**Editorial Comment**

In patients with agitated intensive care unit (ICU) delirium receiving night‐time dexmedetomidine sedation, this study assessed a novel, simplified EEG monitoring device with dedicated software to monitor slow waves as a surrogate for recovering sleep. Sleep quality was generally poor as assessed by both the presence of slow waves and the Richards‐Campbell Sleep Questionnaire, though the two assessment methods were not strongly concordant. The study corroborates previous findings of poor sleep quality in patients with ICU delirium, and the challenges for assessing sleep quality in these patients.

## 1 INTRODUCTION

Delirium is as a generalized dysfunction of cerebral cortical processes with disturbed sleep‐wake cycle, disorientation, and attention deficits. The pathophysiological mechanism of delirium is poorly understood, but inflammation, multiple organ failure, use of benzodiazepines, and sepsis are known risk factors for intensive care unit (ICU) delirium.^1^, ^2^, ^3^ ICU delirium is related to undesirable outcomes such as prolonged hospitalization, increased costs, higher mortality, and long‐term cognitive impairment.^4^, ^5^, ^6^

Sleep disturbances are considered to be important risk factors for delirium.^7^ Diminished total sleep time and reduced non‐rapid eye movement (NREM) sleep are associated with delirium.^8^ Loss of stage N2 electroencephalogram (EEG) features (K‐complexes and spindles) also are common in critically ill patients with delirium and associated with more severe encephalopathy and higher odds of death.^9^ These studies suggest a crucial role for NREM sleep in avoiding and recovering from ICU delirium. In healthy volunteers, dexmedetomidine promotes NREM sleep and produces a state closely resembling physiological sleep in humans, activating normal NREM sleep‐promoting pathways.^10^, ^11^

Slow waves are the most important EEG features of NREM sleep.^12^, ^13^ Originating from the oscillatory activity of neurons in the neocortex and thalamus, slow‐wave activity (SWA) can be measured from the EEG with its power focused below 1 Hz.^14^ Because it is associated with the deep recovering stages of sleep, SWA offers an objective neurophysiologic measure of sleep quality and quantity. Our previous research shows that EEG SWA can be captured reliably using forehead electrodes in the ICU setting.^15^

In this observational pilot study in patients with hyperactive ICU delirium receiving nighttime dexmedetomidine infusion, we investigated the possibility of quantitatively monitoring nighttime EEG SWA and the association of SWA with the clinical evaluation of sleep. We hypothesized that, in these patients, nighttime EEG SWA would offer an objective quantitative measure for evaluating sleep quality and depth.

## 2 METHODS

This prospective observational study was conducted in a 26‐bed medical‐surgical ICU in a tertiary‐level university hospital in Finland. The study was approved by the Northern Ostrobothnia Hospital district review board (135/2017) and local ethics committee (47/2017). Written informed consent for participation was obtained from the next of kin.

### 2.1 Patients

Between October 2017 and March 2019, we screened consecutive eligible ICU patients of age 18–85 years without neurological or neurosurgical admission. Patients with hyperactive delirium (Intensive Care Delirium Screening Checklist [ICDSC] score ≥4) and not receiving mechanical ventilation were included in the study.^16^ A single ICDSC score ≥4 during the day was enough for inclusion and monitoring was performed during the following night. Hyperactivity was defined as increased psychomotor activity, restlessness, agitation, aggression, hyper alertness, hallucinations or inappropriate behavior. Exclusion criteria were history of dementia, condition preventing delirium assessment, treatment restrictions, an acute neurologic injury or other neurological comorbidity, contraindication for dexmedetomidine administration, isolation because of infection, and lack of availability of EEG recording equipment.

### 2.2 Nighttime sedation

The nighttime sleep of the patients was supported following the ICU's standard protocol with dexmedetomidine infusion (4 μg/ml solution), which was started at 9 p.m. with 0.2 μg/(kg*h) infusion (no bolus) and continued until 7 a.m. The bedside nurse targeted the sedation level by adjusting the dexmedetomidine infusion rate to achieve a Richmond Agitation‐Sedation Scale (RASS) of −3 to −2. RASS score was performed every 2 h and prior to possible propofol infusion. A maximum dexmedetomidine infusion rate of 1.4 μg/(kg*h) was not exceeded following the ICU's standard practice. If needed for an adequate level of sedation, propofol was combined with dexmedetomidine with a dose of 1–4 mg/(kg*h). Other sedatives, analgesics, melatonin, and antipsychotics were administered if needed at the discretion of the attending intensivist. Nursing disturbances (taking of laboratory samples, changing of patient position, and changes in drug, fluid, or nutrition administration) between 12 a.m. and 5 a.m. were recorded. The ICDSC score was also obtained 2 h after the discontinuation of dexmedetomidine infusion in the morning.

### 2.3 Clinical data acquisition

All clinical data were input into the ICU electronic clinical data management system (Centricity Critical Care*(8.1) SP7 (8.17.034); GE Healthcare, Barrington, IL, USA). Patient age, sex, body mass index, reason for ICU admission, severity of illness score (Acute Physiology and Chronic Health Evaluation II [APACHE II]) and need for vasoactive or sedative agents were retrieved. Length of stay in the hospital and in the ICU as well as days on mechanical ventilation were also obtained, and the need for additional sedatives or antipsychotics was recorded. Outcomes after 3 months following the hospitalization were obtained via telephone contact and defined using the cerebral performance category score. Good recovery was defined as a score of 1 or 2.

### 2.4 Sleep evaluation

For objective evaluation of sleep, an overnight (from 9 p.m. to 7 a.m.) EEG recording was carried out. The measurements were performed with a disposable BrainStatus electrode (Bittium, Oulu, Finland) placed on the forehead and a wireless BrainStatus amplifier (Bittium, Oulu, Finland) providing a 10‐channel (Fp1, Fp2, Af7, Af8, F7, F8, Sp1, Sp2, T9, T10) EEG recording. The SWA, used as an indicator of recovering deep sleep, was evaluated by offline calculation of the C‐Trend Index (v. 1.0.0.0; Cerenion, Oulu, Finland). C‐Trend is a CE marked medical device software that utilizes artificial intelligence in producing a parameter, C‐Trend Index, of the SWA in EEG. The parameter ranges from 0 to 100 in values, values above 80 referring to high normal SWA and values below 50 referring to abnormal or low SWA.^17^ The software automatically rejects data sequences it considers to contain too much noise. The average index value calculated over the entire recording was used to indicate the average SWA. Furthermore, the percentage of SWA was calculated as the ratio of the duration of recordings corresponding to index values >50 to the duration of the entire recording. The recordings with an average and percentage of SWA < 50 were categorized as representing poor SWA. As the C‐Trend Index was calculated offline after the recordings, the staff was blinded to it during the treatment of the patients. No other devices such as polysomnogram were used for sleep evaluation.

In addition to the objective quantitative evaluation using EEG, the night shift nurse that spent the night bedside also clinically evaluated sleep at 6 a.m. using the Richards‐Campbell Sleep Questionnaire (RCSQ).^18^ For this analysis, the RCSQ parameters “sleep depth” and “sleep quality” were used, with a range from 0 to 100 for each parameter, with 100 corresponding to deep and good‐quality sleep. Scores <70 were considered to indicate poor sleep quality and sleep depth.^19^

### 2.5 Statistical analysis

The study was designed to be observational and a convenience sample size was enrolled. Summary measurements are expressed as means with standard deviations or medians with 25th–75th percentiles. Proportional data are expressed as rates (counts) and percentages. Bivariate comparison of factors related to sleep (SWA and RCSQ parameters) and delirium/clinical data was performed using the Mann–Whitney *U* test. Linear regression models were fitted to the data to investigate the correlation between the SWA and RCSQ parameters, and *R* ^2^ was used to evaluate the goodness of fit. The statistical analysis was performed with the Statistics and Machine Learning Toolbox of MATLAB R2018a software (Natick, MA). Two‐tailed *P* values are reported, and a value <0.05 was considered statistically significant.

## 3 RESULTS

**FIGURE 1:**
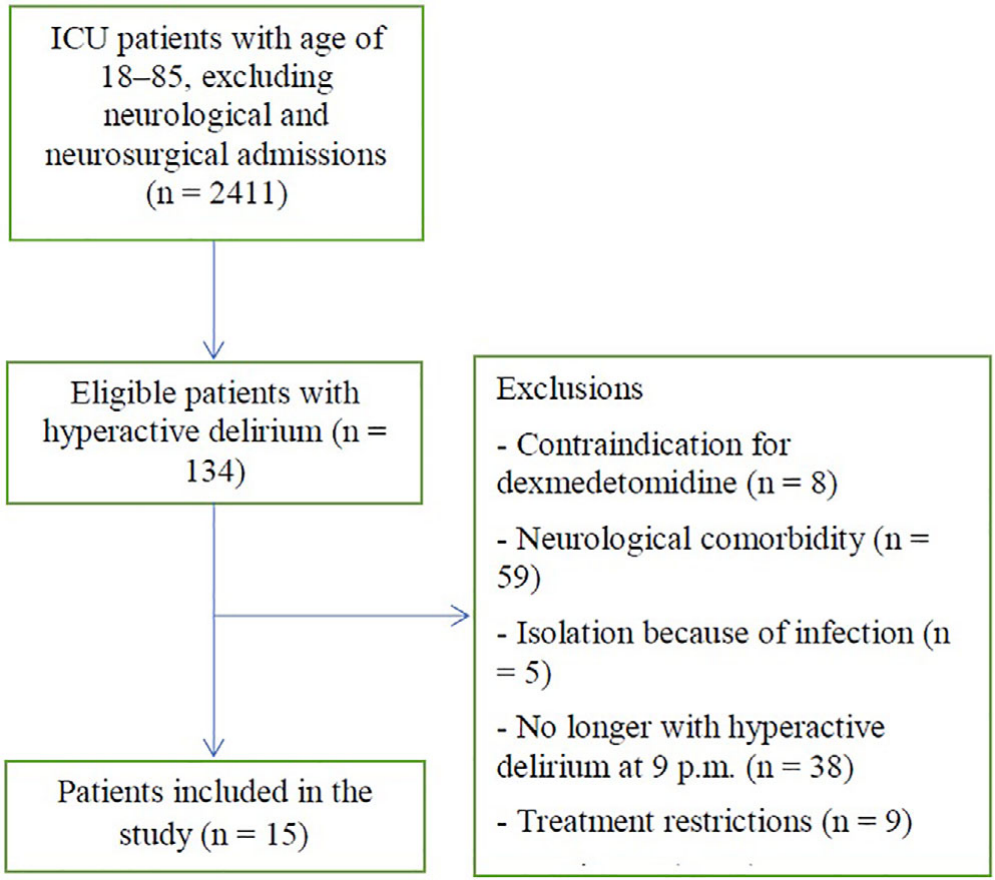
Patient flow chart

During the study period, we had 134 eligible patients with hyperactive delirium, of whom 15 were included in the study (Figure 1). Four of the patients were treated in a single‐patient room. The main reason for exclusion was neurological comorbidity. The demographics and clinical data for the patients are described in Table 1. Most patients were male. One patient died during the ICU stay due to influenza, myocarditis, and multi organ failure 31 days from ICU admission. Rest of the patients experienced a good neurological recovery during the 3‐month follow‐up period.

**TABLE 1:** Demographic and clinical data expressed as mean (SD), median [25th–75th percentiles], or *n* values with percentages

Parameter	All patients (*n* = 15)
Age, years	59 (15)
Sex, male, *n* (%)	9 (60)
Body mass index	30 (7)
APACHEII score on admission	18 [11–21]
Time in ICU prior to the study, days	2.4 [1–6]
Time in mechanical ventilation prior to the study, days	0.49 [0.15–6]
Emergency admission, *n* (%)	11 (73)
Operated, *n* (%)	6 (40)
ICU diagnosis	
Cardiac or aortic surgery, *n* (%)	4 (27)
Pancreatitis, *n* (%)	2 (13)
Pneumonia, *n* (%)	4 (27)
Sepsis, *n* (%)	5 (33)
Total ICU length of stay, days	5.7 [4–9]
Total hospital length of stay, days	15 [12–26]
Good recovery at 3 months (cerebral performance category score <3)	14/15
RASS during EEG monitoring	−3 [−3–‐2]
ICDSC score before dexmedetomidine infusion	5 [4–6]
Total dexmedetomidine dose, μg/kg	5.2 [3–7.8]
Dexmedetomidine maximum dose, μg/kg/h	0.78 [0.54–1.2]
Duration of dexmedetomidine infusion, min	583 [527–587]
Other sedative or psychotropic drugs	
Benzodiazepine, *n* (%)	1
Haloperidol, *n* (%)	2
Propofol, *n* (%)	3
Vasopressor maximum dose during EEG recording, μg/kg/min	0 [0–0.05]
Treatment events disturbing sleep	10 [7–11]
SWA, average	41 [16–67]
SWA, percentage	29 [3–92]
RCSQ questionnaire	
Sleep depth	63 [47–73]
Sleep latency	40 [28–75]
Awakening	60 [29–72]
Returning to sleep	67 [31–80]
Sleep quality	61 [50–70]
Noise	76 [65–92]

The median RASS during the dexmedetomidine infusion was −3 and all patients achieved the predetermined RASS level during EEG monitoring. The median maximum dexmedetomidine dose was 0.78 μg/kg/h. Propofol was combined with dexmedetomidine in three cases. The patients were disturbed during the night by nursing activities for a median number of 10 times. The morning ICDSC score following dexmedetomidine infusion was <4, not fulfilling the criteria for ICU delirium in 53% of the patients. The total nighttime dexmedetomidine dose was significantly higher in those who did not have delirium the following morning than in those with delirium (7.4 μg/kg [4.7–7.9] vs. 4.0 μg/kg [2.9–5.2]; *p* = 0.041). There were no differences between the groups in the median duration of dexmedetomidine infusion (582 min [469–587] vs. 584 min [526–588], *p* = 0.619) or the ICDSC score before the dexmedetomidine infusion (4.5 [4, 5] vs. 5 [5, 6], *p* = 0.282). There was also no differences in the average or percentage of SWA between the groups (42 [16–58] vs. 28 [7–41], *p* = 0.613 and 28 [6–66] vs. 29 [3–96], *p* = 0.867, respectively). The sleep depth and quality did not differ either (67 [42–76] vs. 62 [47–70], *p* = 0.613 and 65 [53–81] vs. 52 [35–70], *p* = 0.536).

**FIGURE 2:**
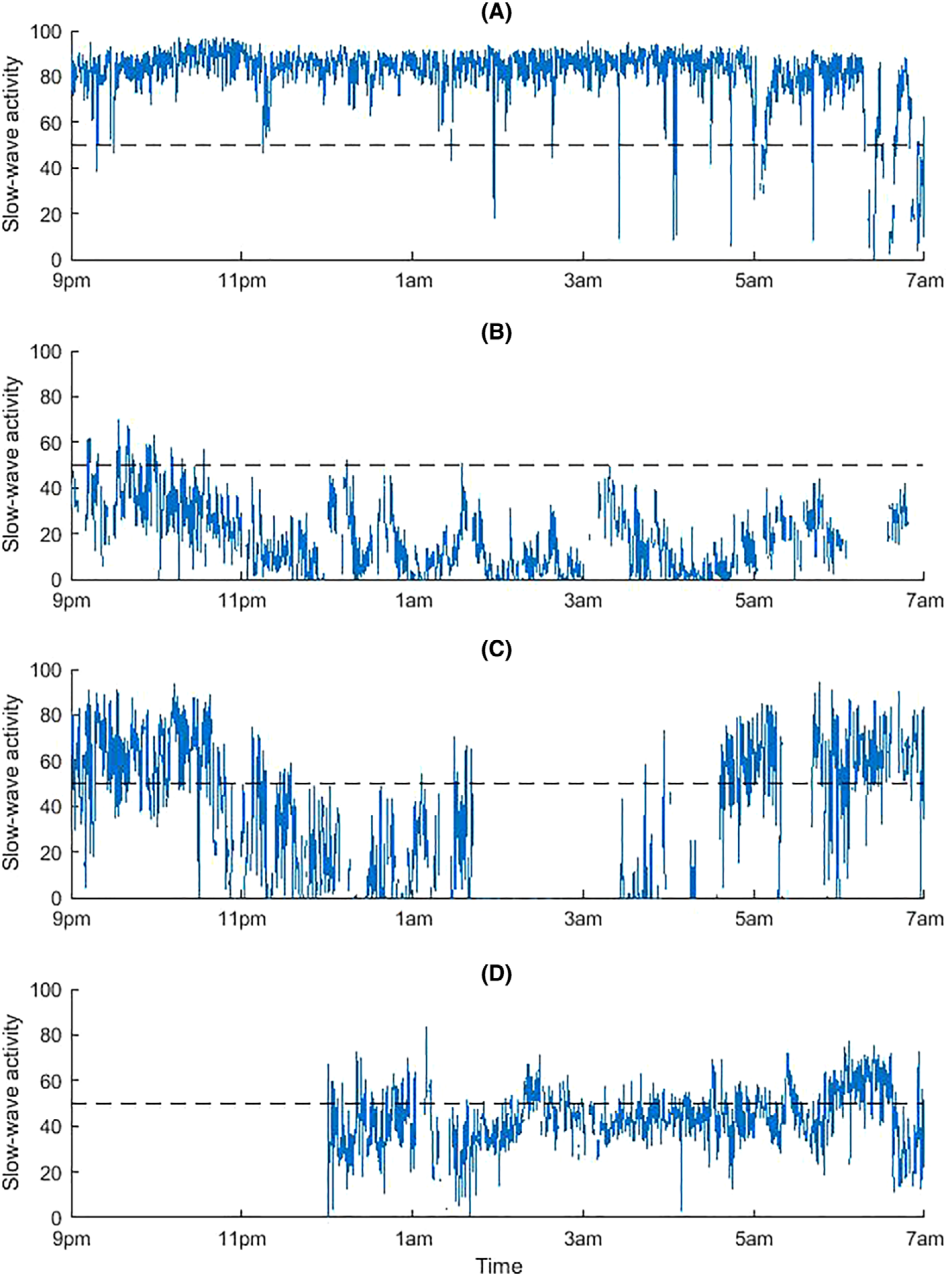
Examples of slow‐wave activity (SWA), that is, C‐Trend Index values, for four patients during the nighttime EEG recording. (a) High SWA during almost the entire recording period. The RCSQ scores for sleep depth and quality were both 70. (B) Low SWA during almost the entire recording period. The RCSQ scores for sleep depth and quality were 40 and 60, respectively. (C) High variability in SWA representing periods of rather high activity in the beginning and end of the recording period, as well as low activity in between. A part of the trend is missing because of poor signal quality. The RCSQ scores for sleep depth and quality were 24 and 14, respectively. (D) Borderline SWA during almost the entire recording period. The beginning of the trend is missing because of poor signal quality. The RCSQ scores for sleep depth and quality were 71 and 61, respectively.

The analysis of nighttime EEG SWA revealed that individual sleep structures varied fundamentally among patients. Figure 2 gives examples of the behavior of SWA, that is, the C‐Trend Index value, for four patients. Overall, the SWA was categorized as poor in 67% of the patients. Sleep quality was poor in 67% and sleep depth was poor in 60% of the patients according to RCSQ. The SWA‐based classification aligned with the RCSQ‐based sleep quality in 87% and with RCSQ‐based sleep depth in 67% of cases.

**FIGURE 3:**
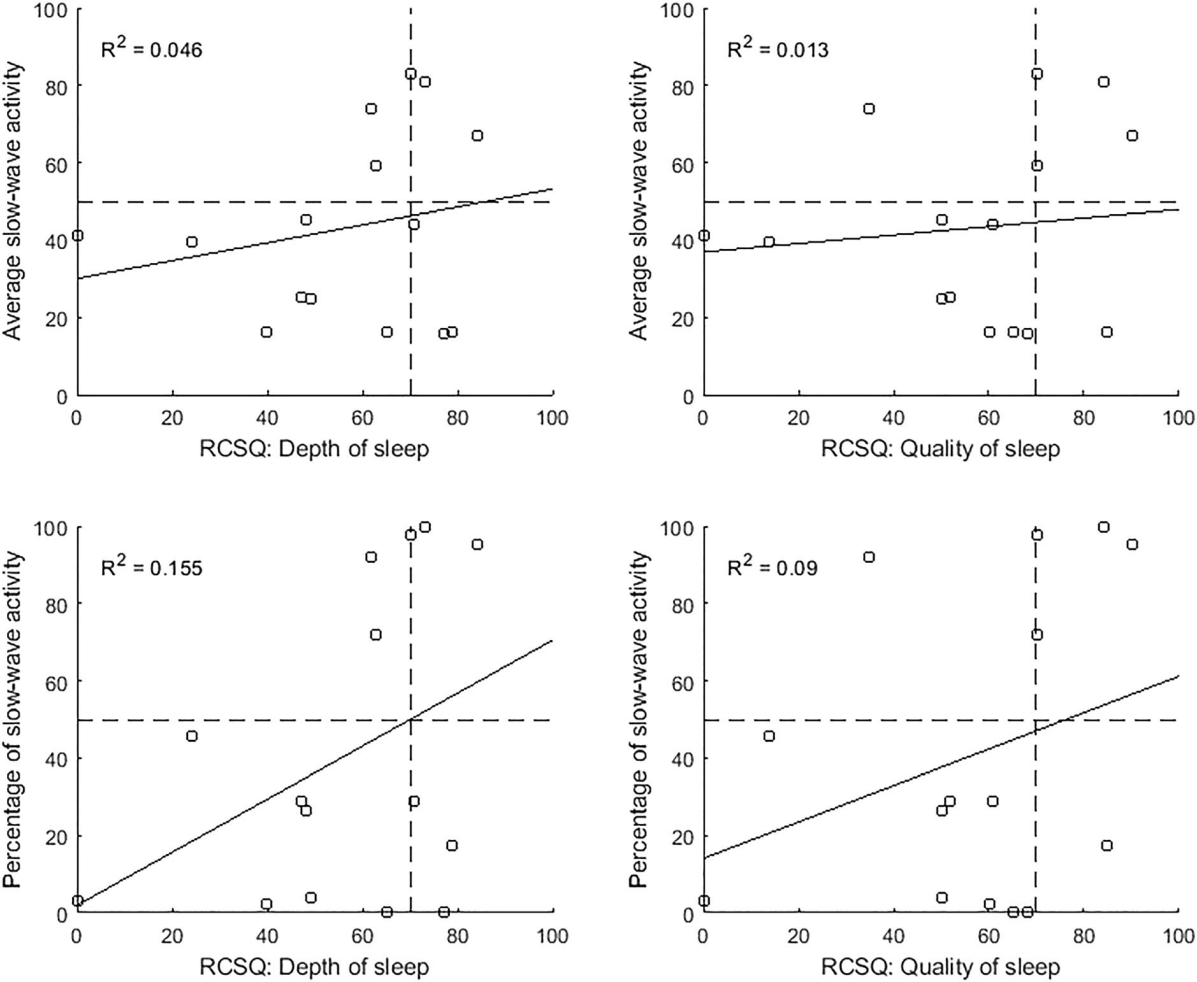
Average slow‐wave activity (SWA) and percentage of SWA, as calculated from C‐Trend Index values, compared with the RCSQ‐based sleep depth and sleep quality scores for each patient (*n* = 15).

Figure 3 shows a comparison of the average SWA and percentage of SWA to the RCSQ scores for sleep depth and sleep quality. The correlation is illustrated with linear regression, and *R* ^2^ is used to evaluate goodness of fit. As the figure shows, *R* ^2^ values were low, indicating poor correlation between the SWA and RCSQ scores as continuous variables. The difference can be especially noted in patients whose sleep was clinically evaluated as deep or high quality but whose SWA value was categorized as poor (lower right quarter of plots in Figure 3). This discrepancy suggests an overestimation of the amount of recovering sleep by the nurse. Average or percentage of SWA did not correlate with total dexmedetomidine dose, dexmedetomidine maximum dose, time in ICU, or time on mechanical ventilation before the study. These values also did not correlate with age, treatment events disturbing sleep, or APACHE II score at admission (data not shown).

## 4 DISCUSSION

This study was performed to investigate quantitative monitoring of nighttime EEG SWA and its association with the clinical evaluation of sleep in patients with hyperactive ICU delirium treated with dexmedetomidine. For SWA measurement, a disposable forehead EEG electrode and a wireless recording device were used in combination with offline calculation of the C‐Trend Index. Our key results are as follows: nighttime sleep, despite being supported with dexmedetomidine infusion, was considered to be poor in most (67%) patients based on both the SWA‐ and RSCQ‐based methods. Furthermore, the SWA and RSCQ evaluations did not align in categorizing sleep quality and depth. Typically, sleep was clinically evaluated to be deep and high quality despite a low SWA value, suggesting overestimation of recovering sleep by the nurse. The detailed analysis of SWA revealed individual and variable sleep structures among patients and offered a potential target, for example, for individual drug dosing to improve the nighttime sleep.

Our findings suggesting suboptimal sleep despite dexmedetomidine infusion are in line previously reported results. In one earlier study, nighttime dexmedetomidine improved sleep by increasing sleep efficiency and stage N2 sleep in patients who were critically ill.^20^ However, only half of the patients in that study slept more than 50% of the night, despite dexmedetomidine administration. In another report, the median percentage of stage N2 sleep with dexmedetomidine infusion was 44% in non‐cardiac surgical patients in the ICU.^21^ Another group assessing 10 patients receiving mechanical ventilation found that sleep efficacy was 50% of the nighttime period during dexmedetomidine infusion, with slow‐wave sleep in only three patients and rapid eye movement sleep in only two.^22^ These findings suggest that, while dexmedetomidine could improve sleep, the results might still be suboptimal if the administration is based entirely on clinical signs or fixed infusion. Here, we found no significant correlation between SWA and dexmedetomidine dosing. Similarly, in non‐intubated patients without delirium in another study, although nighttime dexmedetomidine infusion induced the N2 stage sleep pattern in 69%, dexmedetomidine dosing did not correlate with sleep stage duration.^23^ Our current results and those of these previous studies suggest the need for an objective evaluation of sleep that enables individual adjustment of dexmedetomidine dose to achieve adequate sleep depth and quality to avoid under‐ or overdosing. A good candidate to address this need would be a practical EEG‐based solution.

Of interest, in our series, the total administered dexmedetomidine dose was higher in patients without delirium the next morning, suggesting a beneficial effect unrelated to sleep. There was no statistically significant difference in sleep depth, sleep quality, average of SWA, or percentage of SWA between those with or without delirium in the following morning. We note that the role of sleep disturbance in the development of ICU delirium is not undisputed. In contrast to EEG‐based studies, polysomnography studies have yielded contradictory results regarding the association between disturbed sleep and development of delirium.^24^, ^25^ Many other pathophysiologic mechanisms contribute to the development of delirium and could be affected by dexmedetomidine.^26^ Furthermore, reduced delirium risk with dexmedetomidine may arise from avoidance of benzodiazepines.^27^ In our series, the only patient who also received diazepam experienced both low SWA and poor sleep quality according to the RCSQ.

We also found a discrepancy between the clinical evaluation of sleep based on RCSQ parameters and the EEG SWA. To our knowledge, this has not been studied earlier in patients suffering from ICU delirium. However, in a wider sense, subjective perception of sleep has been reported to be inconsistent with objective EEG indicators of sleep^28^ as well as sleep evaluation based on polysomnography.^29^ Furthermore, in intensive care, nurses tend to give higher ratings on the RCSQ compared with patient‐reported outcomes, suggesting overestimation of sleep quality.^30^ A recent randomized controlled trial showed that although dexmedetomidine administration reduced the incidence of delirium, it did not affect patient‐reported sleep quality.^31^ Taken together, questionnaires do not seem to be sufficiently robust for reliable assessment of sleep quality and quantity.

All patients in the current study were disturbed at least once during the night by treatment events, but SWA did not correlate significantly with the number of disturbances. We did not quantify the effect of environmental factors in the ICU, such as noise and lighting. Others have shown that patient sleep in the ICU is disrupted by noise, light, and treatment events.^32^, ^33^, ^34^ We also note that clinical assessment of sedation or sleep can disturb the patient and interrupt sleep, which also argues for easy‐to‐use bedside technology for sleep evaluation. Indeed, monitoring could even be used to alert the bedside nurse to delay unnecessary treatment events when the patient is in deep sleep states.

The current study has several limitations. First, we recorded only one nighttime period in a relatively small number of patients without a control group. A longer study period with more patients and would be needed for more conclusive findings. Small sample size might be the reason for non‐significant results in the statistical comparisons which should be taken into account when interpreting the results. Furthermore, a continuous 24 h EEG recording would be preferred to evaluate the differences between wakefulness and sleep as well as the effect of nighttime and daytime findings. This would also help in evaluating the possible effects of delirium on the EEG background activity and differentiate those from the changes caused by the natural sleep. Comparison of SWA in patients with and without delirium would also be beneficial in future. The sedatives might also have played a role in the current results considering that, in addition to the dexmedetomidine, propofol was used in 20% of the patients. However, this investigation was designed as a descriptive pilot study, and the results should be regarded as hypothesis generating.

Further studies are needed to show whether nighttime SWA correlates with hyperactive delirium and if EEG‐guided dexmedetomidine dosing could be used to alleviate the condition. The first step could be to investigate whether the amount of nighttime SWA could be increased with individual EEG‐guided administration of dexmedetomidine. We hypothesize that this intervention could halve the number of patients experiencing poor SWA, that is, reduce the incidence from the 67% observed in this study to 33%. To confirm this possibility would require a study of at least 66 patients allocated equally between the intervention and control groups (alpha = 0.05; beta = 0.2; power, 0.8). The C‐Trend Index that we used for quantitative evaluation of SWA should be further investigated in patients with hyperactive delirium as well as natural sleep. For example, the optimal threshold value of the index for indicating sufficient depth of sleep is currently unclear, as is what the required duration would be for exceeding this threshold to guarantee adequate sleep for a single night. The SWA derived from the C‐Trend Index should be validated in healthy adults through a comparison to polysomnography, which remains the reference standard for studying sleep.^35^ Further studies could also address other sleep macro and microstructures and how they are affected by delirium.

According to both EEG‐based SWA values and clinical evaluation based on RCSQ, the quality and depth of nighttime sleep are poor in most patients with hyperactive delirium despite supportive dexmedetomidine infusion. Furthermore, categorizations of sleep based on RCSQ scores and SWA values did not align. The findings highlight the need for an objective sleep evaluation and individual drug dosing in the intensive care setting to improve sleep for patients with delirium.

## CONFLICT OF INTEREST

Jukka Kortelainen is a co‐founder of Cerenion Oy, Oulu, Finland. All other authors have no conflict of interest.
